# Peer review: Together we can make it work

**DOI:** 10.5271/sjweh.4233

**Published:** 2025-05-01

**Authors:** Ute Bültmann, Cécile RL Boot

**Affiliations:** Department of Health Sciences, Community and Occupational Medicine, University Medical Center Groningen, University of Groningen, Groningen, The Netherlands. E-mail: u.bultmann@umcg.nl; Amsterdam Public Health Research Institute, Department of Public and Occupational Health, Amsterdam UMC, VU University, Amsterdam, The Netherlands. E-mail: crl.boot@amsterdamumc.nl

Scientific journals, like the *Scandinavian Journal of Work, Environment and Health*, are dependent on their peer reviewers. Without a rigorous peer review of submitted papers, there are no publications and no journals. As researchers, we know that a critical and constructive evaluation of research in the review process is essential to move the science in our field forward. While worldwide the academic peer review system is under pressure (1–3), a rigorous peer review is more important than ever before for three reasons we highlight below: (i) an exponential increase in scholarly journals; (ii) the reduced reliance on facts; and (iii) the intensification of work.

**Figure 1:**
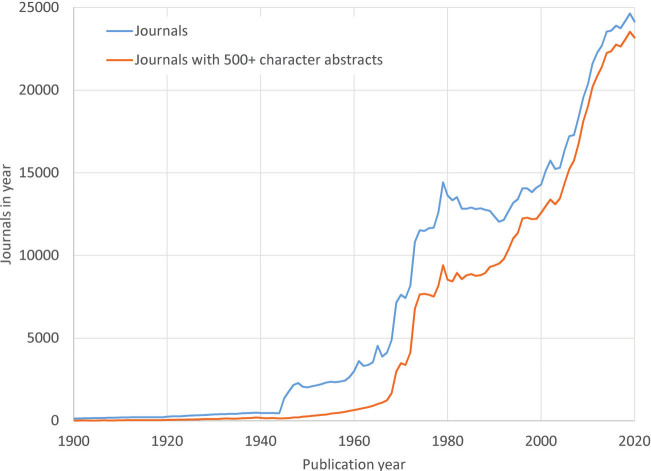
Number of different journals in Scopus by year (4)

In the past years, researchers have received increasingly review requests from what seems a sheer flood of journals from all over the world. As shown in figure 1, the number of scientific journals has increased exponentially (4). Within this increase, we see robust scientific journals but also predatory ones, often characterized by high article processing charges, false claims of peer review, and the unethical listing of academics (or even fake scholars) on editorial boards without their consent (5). To date, www.predatoryjournals.org – a website that provides information and resources on predatory publishing practices – lists 1363 publishers and 2780 journals. Moreover, artificial intelligence (AI) technologies make it increasingly difficult for researchers and society to distinguish between facts (ie, evidence) and fiction. Robust peer reviews and high-quality journals are thus extra important nowadays. The proper use of AI in the writing, reviewing and publishing process is for all of us, editors, reviewers and researchers alike, a new challenge we have to carefully address. Clear guidelines of when AI can or cannot be used are in continuous development (see among others the BMJ www.bmj.com/content/ai-use). Researchers use AI tools for support in writing introductory paragraphs and perhaps in other parts of studies. This proliferation of AI tools will likely result in an increased number of papers. At the same time, we would like to stress that the *Scandinavian Journal of Work, Environment and Health* prohibits reviewers from uploading manuscripts to AI platforms for review purposes as this clearly breach confidentiality, thus the availability of AI tools will likely be only of limited help in the review process.

In addition to the tsunami of submitted papers, finding peer reviewers is another bottleneck in the academic peer review system. This is likely related to the intensification of work over the past decades. Society has become more globalized and digitalized, both increasing work pace, with major implications for the 24/7 work environment, making the working life more demanding and intense (6). Academics are experiencing high levels of work pressure (7), which can reduce their availability and willingness to participate in peer reviews (2). Yet, at a time when science is under increasing global pressure—from the rise of misinformation to sweeping policy changes and budget cuts—the need for skilled peer reviewers has never been greater. A robust and thorough review process is essential to uphold research quality in these changing times (8). To achieve this, we must build a large and diverse pool of reviewers that reflects the full breadth of researchers and research in our field.

With this editorial, we would like to send out a call for peer review skills development in a mentor–mentee relationship. We at the Journal, but also the entire research community, need the next generation(s) of researchers to learn and apply peer review skills as we strongly believe they are crucial for good science. Senior researchers have many years of valued reviewing experience, and it is exactly this expertise they can pass on to their early-career colleagues. We propose to prioritize mentor–mentee relationships and approach performing a peer review as a necessary cornerstone of the competence and skill development of emerging researchers. As the peer-review process is often not taught by graduate schools or PhD programs, it is the responsibility of the individual PhD supervisor or research leader to teach or promote the important skill of providing constructive feedback which is really different from writing a scientific paper. We recognize that mentoring a new reviewer requires time and commitment from both the mentor and the mentee, as it is a learning process. However, this investment will pay off by helping to cultivate the next generation of thoughtful, critical, and constructive peer reviewers. Reviews conducted through a mentor–mentee collaboration may also lead to higher-quality outcomes, as each reviewer brings a fresh perspective—and no single reviewer can catch everything. In a time of growing societal misinformation, peer review must remain a high priority to safeguard the integrity of research. When it comes to the peer-review process itself, numerous checklists and guidelines exist, but our main message and call for action is to prioritize peer review tasks. In particular, we encourage early-career researchers to collaborate with their senior colleagues in the peer-review process. In 2024, Fagher & Verhagen (9) insightfully summarized the advantages of early-career researchers contributing to peer review. Peer review is an important academic skill that should be incorporated into eg, PhD portfolios. The *Scandinavian Journal of Work, Environment and Health* provides early-career researchers with the opportunity to conduct peer reviews under the responsibility of a senior researcher. We hope this editorial contributes to our peer review system by motivating senior researchers to mentor early-career researchers, and by stimulating early-career researchers to develop their peer review skills. Especially, we hope that all our readers are now extra eager to prioritize peer review tasks and designate their precious time for peer review in good-standard journals.

Together we can make it work!
